# Pharmacological Properties of Parasitic Plants: Current Evidence and the Role of Parasitic Lifestyle

**DOI:** 10.3390/plants15091359

**Published:** 2026-04-29

**Authors:** Tzvetelina Zagorcheva, Denitsa Teofanova, Mariela Odjakova, Junmin Li, Lyuben Zagorchev

**Affiliations:** 1 Research and Development and Innovation Consortium, 111 Tsarigradsko Shose Blvd., 1784 Sofia, Bulgaria; tzvetelina.zagorcheva@gmail.com; 2 AgroBioInstitute, Agricultural Academy, 8 Dragan Tsankov Blvd., 1164 Sofia, Bulgaria; 3 Faculty of Biology, Sofia University “St. Kliment Ohridski”, 8 Dragan Tsankov Blvd., 1164 Sofia, Bulgaria; teofanova@biofac.uni-sofia.bg (D.T.); modjakova@biofac.uni-sofia.bg (M.O.); 4 School of Life Sciences and Health, Huzhou College, No. 999 Miaofengshan North Road, Huzhou 313000, China; lijmtzc@126.com; 5 Zhejiang Key Laboratory for Restoration of Damaged Coastal Ecosystems, School of Life Sciences, Taizhou University, No. 1139 Shifu Road, Taizhou 318000, China

**Keywords:** alkaloids, biologically active compounds, parasitic plants, phenolics

## Abstract

Parasitic plants represent a unique group of angiosperms that extract nutrients from host plants through specialized structures called haustoria. With over 4750 recognized species, these plants vary in their dependence on hosts, classified as holoparasites (completely non-photosynthetic) or hemiparasites (partially photosynthetic). Despite their parasitic lifestyle, these plants contribute significantly to ecological stability by regulating plant communities. Some parasitic species, such as *Striga* and *Orobanche*, are major agricultural pests, while others, including *Cistanche* and *Cynomorium*, are valued for their medicinal properties. Parasitic plants in general are rich in secondary metabolites with potential pharmacological significance. These compounds, including alkaloids, phenolics, and terpenoids, display antimicrobial, anticancer, and immunomodulatory effects. Mistletoe (*Viscum album* L.) produces lectins and viscotoxins, which exhibit cytotoxic and immune-stimulating properties. Traditional medicine has long utilized parasitic plants, and modern pharmacological research continues to uncover their potential in drug development. However, an intriguing question arises: whether they are superior in any way to their non-parasitic counterparts, or just received more attention due to their unique appearance. Understanding the unique chemistry of parasitic plants provides insights into their ecological role and offers opportunities for advancements in medicine and agriculture.

## 1. Plant-to-Plant Parasitism—Basic Knowledge

Parasitic angiosperms are an intriguing group of plants that parasitize other plants. Currently, there are more than 4750 recognized species of parasitic plants belonging to 292 genera [1]. What makes these plants different is their ability to extract nutrients from host plants through a vascular element’s connection, established through a specialized organ called a haustorium, which is common for all parasitic plants [2]. Depending on the degree of specialization, parasitic plants may have completely lost their photosynthetic ability, or, to the contrary, may be fully photosynthetic [3]. Based on this, and preferably over the terms obligate and facultative, parasitic plants are divided into holoparasites (non-photosynthetic) and hemiparasites (photosynthetic) [4]. Furthermore, depending on the organ invaded, parasitic plants can be root or stem parasites.

Nickrent [1] recognized 12 lineages of parasitic angiosperms, among which the most important in terms of species number are the sandalwoods (Santalales, stem hemiparasites, over 2400 species), dodders (Solanales, single genus of stem holoparasites, over 200 species), and the family Orobanchaceae (Lamiales, root hemi or holoparasites, single family with over 2000 species). It should be noted, however, that dodders do have a certain degree of photosynthetic ability [5]. Dodders, along with the *Striga* and *Orobanche* genera of the Orobanchaceae family, are among the most noxious weeds and agricultural pests worldwide, causing losses of hundreds of millions of USD [6] due to severe reductions in crop yields.

Other parasitic plants, however, are unique and intriguing encounters. The well-known *Rafflesia* spp. represent a genus of endemic and endangered plants from the tropical forests of South-East Asia, having the largest known flowers, contrasting the smallest vegetative body among plants [7]. *Cynomorium coccineum* L. and *C. songaricum* Ruprecht (probably a single species) are holoparasitic halophytes with bizarre looks, distributed from the Mediterranean to far eastern Asia [8]. From an ecological point of view, parasitic plants are not pests at all. On the contrary, they are important functional regulators of plant communities and of ecosystem functioning [9,10,11].

What is really special about parasitic plants, however, is the vascular connection with the host plants, allowing extensive transfer of essential nutrients, but also macromolecules and secondary metabolites. This connection suggests that their metabolic profile is largely shaped by the host plant, which contributes to their pharmacological properties.

## 2. Parasitic Plants—From Traditional Medicine to Modern Application

Parasitic plants have played a significant role in ethnopharmacology for centuries. Across various cultures, these plants have been utilized for their diverse bioactive compounds, which exhibit antimicrobial, anti-inflammatory, analgesic, and anticancer properties [12,13,14,15,16,17,18]. These plants have been incorporated into traditional medicine across different cultures for centuries, and modern research is now beginning to validate and explore their pharmacological benefits.

**Figure 1:**
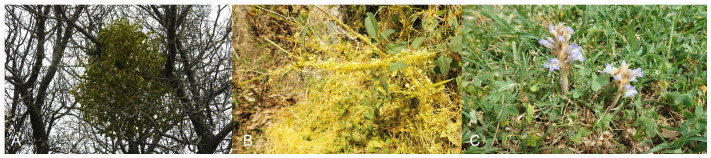
Examples of parasitic plants. (**A**) *Viscum album*; (**B**) *Cuscuta platyloba* Progel.; (**C**) *Orobanche* sp.

One of the most well-known parasitic plants in ethnopharmacology is *Viscum album* (European mistletoe, Figure 1A). Used in traditional European and Asian medicine for centuries, mistletoe extracts have been employed to treat a variety of diseases [19,20,21,22,23]. Various uses include treating hypertension and atherosclerosis, bone and joint disorders, headache, immune system stimulation, and as a sedative [20]. In Northern India, the plant is also used to lower blood pressure, ease anxiety, promote sleep, and treat epilepsy, as reported in Poland. Numerous reports demonstrated that *V. album*, along with other mistletoes, is particularly rich in flavonoids, phenolic acids, lectins, and viscotoxins [22,24]. While flavonoids are responsible for a variety of pharmacological effects, lectins and viscotoxins are associated with anti-cancer activities due to their specific binding to carbohydrate epitopes on tumor cells, leading to agglutination, apoptosis, and inhibition of angiogenesis and cell proliferation [25]. The types of accumulated lectins are strongly dependent on the host trees [22], which in turn may affect the pharmacological properties. The molecular mechanisms of action of mistletoe bioactive compounds are probably the best understood among parasitic plants and have recently been reviewed in detail [26]. For example, mistletoe extracts induce apoptosis mainly by inhibiting AKT phosphorylation, upregulating proapoptotic proteins and activating caspases, and by inducing cell cycle arrest through enhanced p38 and JNK1 activation and downregulation of cyclins and cyclin-dependent protein kinases.

Some of the claimed activities were further confirmed in pre-clinical experiments in rats and mice [19,20,27]. In contemporary medicine, mistletoe preparations, particularly Iscador [28] and Helixor [23], are used as adjunct therapies in cancer treatment and have been evaluated in clinical trials [23,29,30]. Research suggests that mistletoe extracts may possess immunomodulatory and cytotoxic properties, which could help slow tumor growth and improve the quality of life in cancer patients. Its bioactive compounds, including lectins and viscotoxins, exhibit apoptosis-inducing effects in cancer cells, making it a subject of ongoing clinical investigations [28]. There are also several patents published on mistletoe preparations for treating obesity and hair loss, enhancing immunity, and inhibiting inflammation [24].

Another notable parasitic plant is *Cuscuta* (dodder, Figure 1B), which is widely used in traditional Chinese medicine. *Cuscuta chinensis* Lam. is particularly valued for its purported ability to enhance male fertility, support liver health, and improve kidney function [31,32,33,34]. *Cuscuta* seeds, known as Tu Si Zi, are used to tonify the kidneys and nourish yin and yang energies. At the phytochemical and mechanistic levels, modern studies have identified flavonoids, lignans, and alkaloids in Cuscuta, which are associated with antioxidant, neuroprotective, antidiabetic, antimicrobial, and hepatoprotective activities, as demonstrated in vitro [12,17]. Besides *C. chinensis*, *C. reflexa* Roxb. is a highly studied potent pharmacological plant, and other species have also been shown to exhibit a variety of activities [17].

Advancing to in vivo evidence, *Cuscuta* extracts have been extensively investigated in rodent models, confirming multiple pharmacological effects, including hepatoprotective and metabolic regulatory activities [31,35,36]. These findings are supported by studies in various cell lines, which further elucidate molecular mechanisms [37,38]. At the clinical level, available studies remain limited and are primarily focused on psychological disorders [39,40], indicating that high-level clinical validation of *Cuscuta*’s traditional uses is still incomplete.

In Africa, *Striga* species, commonly known as witchweed, have been employed in traditional remedies for various ailments. *Striga asiatica* (L.) Kuntze and *Striga hermonthica* (Delile) Benth., notorious agricultural parasites [41,42], have been used in folk medicine for treating snakebites, gastrointestinal disorders, and inflammation [43,44]. At the experimental level, extracts from *Striga* plants have demonstrated in vitro antibacterial and antifungal activity [45,46], suggesting potential for antimicrobial drug development. However, in vivo and clinical evidence remain scarce, limiting pharmacological validation.

Similarly, species of the genus Orobanche (broomrape, Figure 1C), which parasitize primarily leguminous crops, have long been utilized in traditional Persian and Middle Eastern medicine for their purported diuretic and anti-inflammatory effects [47,48]. These applications are largely supported by ethnopharmacological evidence, with relatively limited experimental validation to date. Nevertheless, their continued use suggests the presence of bioactive constituents that may be relevant to fluid balance regulation and inflammatory processes.

Within the same family, Orobanchaceae, the genus *Cistanche* (Herba Cistanche, Rou Cong-Rong) is among the most extensively studied and highly valued medicinal groups in Traditional Chinese Medicine [13,49]. Traditionally used for “kidney-tonifying” and anti-aging purposes, *Cistanche* has attracted increasing scientific interest due to its broad pharmacological profile. In contrast to *Striga*, substantially higher levels of evidence are available for *Cistanche*, including well-designed preclinical studies and emerging clinical trials investigating its effects on cognitive performance, neuroprotection, and muscle function [50,51]. These studies suggest potential roles in modulating neurodegenerative processes, enhancing mitochondrial function, and improving physical endurance, although further large-scale clinical validation remains necessary.

From a phytochemical perspective, while Striga species appear to contain predominantly flavonoids and glycosides [52], holoparasitic members of the Orobanchaceae—including *Orobanche* and *Cistanche*—are characterized by a richer and more diverse spectrum of secondary metabolites. These include phenylethanoid glycosides (e.g., echinacoside and acteoside), iridoids, lignans, and various phenolic compounds [53]. Such diversity is often associated with multifunctional pharmacological effects, particularly antioxidant, anti-inflammatory, and neuroprotective activities, which may partly explain the broader therapeutic applications and stronger experimental support observed for *Cistanche* species.

Another significant parasitic plant is *Hydnora africana* Thunb., a subterranean holoparasite native to Africa. Traditionally used to treat diarrhea, dysentery, and skin infections [54], it is supported by ethnomedical evidence and phytochemical studies. Modern studies have confirmed the presence of tannins, flavonoids, and phenolic compounds, which contribute to its antimicrobial and astringent properties [18]. However, pharmacological validation remains largely limited to in vitro studies.

Not much different in lifestyle, *Cynomorium songaricum* is also a valuable medicinal plant in Northern China and Mongolia. Phytochemically, *Cynomorium songaricum* contains a rich array of bioactive compounds, including flavonoids, triterpenes, polysaccharides, and phenolic acids, which contribute to its antioxidant, anti-inflammatory, and immunomodulatory effects [55]. Several studies using rodent models have been published in recent years [56,57]. Nevertheless, most evidence remains preclinical, highlighting the need for further clinical validation.

However, just as with any other plant extract, the use of parasitic plants in contemporary pharmacology is limited by several important limitations. These extracts represent a complex mixture of chemical compounds that are strongly influenced by environmental factors and therefore very difficult to standardize [58]. In addition, the chemical composition of parasitic plants may be highly dependent on their hosts, a point further discussed in the following sections and representing an additional challenge.

**Figure 2:**
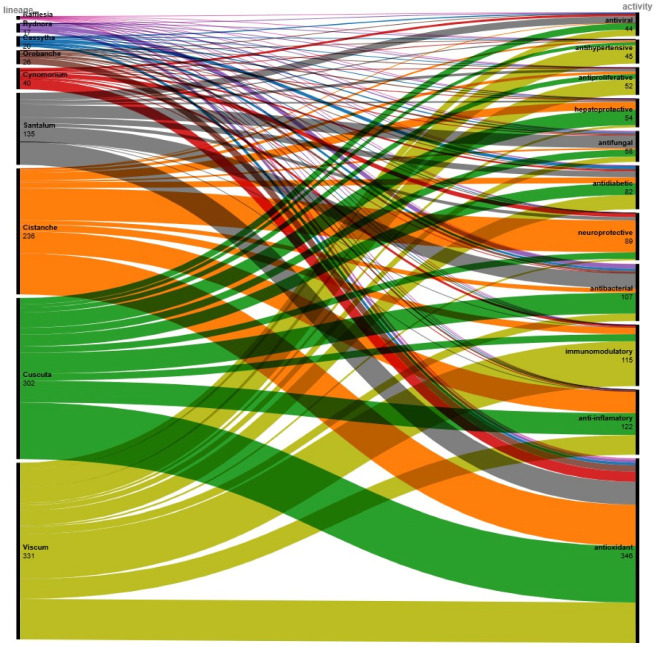
A bipartite diagram illustrating the distribution of reported biological activities across major parasitic plant lineages. The left panel shows the number of documented studies for each genus, while the right panel summarizes the corresponding pharmacological activities. The width of each connecting band is proportional to the number of reports (indexed in Scopus, excluding review papers) linking a given lineage to a specific biological activity.

In a systematic approach towards comparative classification of the pharmacological potential of different parasitic plant lineages, we constructed a bipartite diagram, taking the number of independent reports as weight (Figure 2). The number of papers was taken from Scopus on 02.03.2026, limiting the search to Articles, to subject areas “Medicine” and “Pharmacology, Toxicology and Pharmaceutics”. A search was conducted using genus names + activity, as shown in the figure. Papers reporting more than one activity were not filtered; e.g., they are counted separately for each activity. Only research papers were counted, excluding reviews. The alluvial diagram was constructed using the RAWGraphs online application.

Apparently, the highest number of papers claiming pharmacological activities was for the Viscum and Cuscuta genera, followed by Cistanche and Santalum, while less widely distributed parasitic plant genera like *Rafflesia* and *Hydnora* are relatively poorly studied. There is also a certain uneven distribution: for example, neuroprotective activity was reported almost exclusively for Cistanche spp., with fewer reports for Cuscuta and Viscum, whereas antiproliferative (e.g., anticancer) and Cuscuta and Viscum dominated antihypertensive studies. Therefore, we can assume that different parasitic plant lineages may exhibit different pharmacological activities.

It should be noted that the paper count for each lineage does not reflect the number of unique papers, as some manuscripts report multiple activities. The number shown may also be significantly lower, as not every paper is properly indexed in Scopus (some papers do not fall into the subject areas, although they report pharmacological activities). Most importantly, readers should acknowledge that this diagram is based on the number of scientific papers on the topic and should not be interpreted as a comparative quantitative study of pharmacological activity; e.g., this does not mean that *Cuscuta* spp. has stronger antioxidant activity than *Santalum* spp.

Apparently, there are hundreds, if not thousands, of reports and review papers describing the phytochemistry and pharmacological properties of parasitic plants. Therefore, a major question appears: Are parasitic plants superior in their pharmacological properties to other plants, and if so, what is the common basis of such superiority, considering the non-connected evolutionary pathway toward parasitism in different taxa and the varieties of parasitism? To test this hypothesis, we conducted a review of the available literature to link the characteristics of parasitic lifestyles to the pharmacological properties of the main medicinal parasitic plants. Studies were selected based on their relevance to these themes, with priority given to peer-reviewed articles reporting original experimental data or comprehensive reviews. The major taxa discussed, as well as their pharmacological activities, were selected based on their prevalence in the scientific literature; this is by no means an exhaustive list of parasitic plant species and their activities.

## 3. Special Considerations Related to the Parasitic Lifestyle

### 3.1. The Bizarre Appearance

Parasitic plants, and especially non-photosynthetic holoparasites, are some of the most bizarre-looking plants in the world. In addition, they are comparatively rare. Therefore, they are often a subject of mythologization. For example, *Rafflesia* spp. is a subject of multiple beliefs in the mythology of the indigenous peoples of South-Eastern Asia. In some areas, its ethnobotanical use is associated with the belief that this flower can deter evil spirits [59]. In the Mediterranean region, *Cynomorium coccineum* has been used as a medicinal plant to treat infertility due to its resemblance to a male sexual organ [60]. Dodders (*Cuscuta* spp.) are a subject of multiple superstitions across many countries, which were summarised previously [16,61]. Mistletoes have also been a subject of multiple myths and beliefs since ancient times [62]. Therefore, a major question arises: whether parasitic plants were extensively used in traditional medicinal practices because of their phytochemical features or because they attracted attention for their odd appearance, surrounded by popular beliefs. Although this question is irrelevant in recent times, when chromatography coupled to mass spectrometry enables exhaustive characterization of bioactive compounds, it remains fundamental to the null hypothesis that parasitism is unrelated to medicinal properties and that parasitic plants are no better than other medicinal plants.

### 3.2. The Intrinsic Metabolomic Potential of Parasitic Plants

Parasitic plants in general, and especially holoparasitic plants, such as *Rafflesia*, *Orobanche*, and *Cuscuta*, have undergone extensive genome reduction, including the loss of genes involved in primary and secondary metabolism. This is because they no longer rely on self-sustaining processes such as photosynthesis and nutrient synthesis, instead acquiring essential compounds from their hosts. In the first place, plastids in non-photosynthetic holoparasites are highly degenerated [3,63,64,65], thus depriving the parasites of several important biosynthetic pathways. These include the methylerythritol phosphate (MEP) pathway of terpenoid synthesis [66] and the shikimate pathway, which starts in the plastids and is important for the biosynthesis of polyphenolics and aromatic-acid-derived alkaloids [67,68]. Moreover, the constant reduction of NADP^+^ and the production of ATP, generally needed for biosynthesis and readily generated during the light reactions of photosynthesis [69], should be compensated for by the oxidation of sugars [70,71] in the parasites, which poses a certain limitation. Otherwise put, holoparasitic plants lack a highly effective metabolite factory in the form of chloroplasts. In addition to the above, holoparasitic plants exhibit gene loss across many other pathways, as demonstrated in *Cuscuta* [72]. Therefore, it is not surprising that the diversity of secondary metabolites, determined in many Cuscuta species, was suggested to be acquired and highly dependent on the host plant species [73,74]. The situation appears to be similar among holoparasites of the Orobanchaceae family [75]. However, a more intriguing possibility is that parasitic plants can further chemically modify host metabolites, potentially producing previously unknown compounds with superior or distinct pharmacological activity [75]. Overall, despite their established role in ethnopharmacology, parasitic plants do not appear to be particularly adept at synthesizing bioactive compounds.

### 3.3. The Interaction with the Host

The main common feature of all parasitic plants is the organ of parasitism, collectively called the haustoria [2,76]. Though differences exist among parasitic taxa, this distinct organ, which establishes a physiological connection between the host’s vascular elements and the parasite, is what characterizes a parasitic plant as such. While the function of haustoria is mainly to ensure a constant flow of mineral and organic nutrients, they also serve as an extensive, bidirectional highway for the exchange of various organic compounds, macromolecules, and even viruses [77,78,79].

There are two main considerations here. First of all, the interaction between the parasitic plant and the host involves the manipulation of gene expression, which may be either purposeful (e.g., the parasitic plant adjusts the metabolism of the host to better suit its needs) [80,81], or the host may respond to the parasitism by synthesizing various defensive molecules [82], including secondary metabolites with defensive functions and putative pharmacological significance. Some examples of intentional manipulation include the differential changes in certain terpenoids emitted by *Artemisia campestris* L. in response to *C. campestris* Yunck. parasitism [83], while defense-related increases in host metabolites include an increase in phenylpropanoids in tomato in response to *C. reflexa* [84].

In this respect, we can speculate that parasitic plants would induce the host plant’s overall secondary metabolism, thereby shaping its pharmacological properties. What is more important is that, through the haustorial connection, the parasitic plant would acquire secondary metabolites from the host, which may or may not be further modified; thus, the parasite’s pharmacological properties would depend on the host plant species. Indeed, multiple reports clearly show that the parasitic plant metabolite profile strongly depends on the host range in Cuscuta [85,86] and Orobanche [87], also suggesting significant variations in the pharmacological properties of the parasites. An explicit view, published in *Cuscuta* phytochemistry, but also easily extrapolated to most parasitic plants, claims that the influence of the host plant is so profound that the phytochemical composition of the parasite is entirely dependent, and most of the patents issued on medicinal activities could be proven invalid when the host plant is different [73]. Similarly, the flavonoid profile of *V. album* is strongly dependent on the host tree species [24,88].

Stated, at one extreme, the reduced synthetic ability of parasites, combined with extensive molecular exchange, means that the phytochemical composition is entirely dependent on the host species, implying they lack their own pharmacological capacity. We observed this in our own research, where the phenolic content and antioxidant activity of Cuscuta were strongly affected by host identity [74]. However, this study was based on a single host system, while dodders are known to parasitize multiple hosts from distant taxa simultaneously. Looking again at the flavonoid profile, despite the broad host range and different environmental conditions, the content is relatively similar, allowing for distinction between the two dodder species [89].

However, dodders are not the usual case among parasitic plants in terms of the number of hosts simultaneously affected, as most other representatives in our review are restricted to a single host species. In the case of *V. album*, as well as *Cistanche* spp., the host identity could be easily controlled, thereby eliminating host influence as a factor contributing to phytochemical variability.

## 4. Chemical Background of Pharmacological Properties

Plant bioactive compounds are specialized metabolites that play key roles in plant–environment interactions, including defense against herbivores and pathogens, signaling, and adaptation to environmental stress [90,91]. These compounds are commonly classified into major groups, such as alkaloids, phenolics, and terpenoids, based on their chemical structures and biosynthetic origins [92]. However, in parasitic plants, the distribution, diversity, and functional relevance of these metabolite classes differ markedly from those observed in autotrophic species, reflecting their unique biology and dependence on host plants.

Among the major classes of secondary metabolites, phenolic compounds—particularly flavonoids and phenolic acids—are the most consistently reported and widely distributed in parasitic plants. These compounds have been identified across a broad range of taxa, including *Viscum*, *Cuscuta*, *Striga*, *Orobanche*, and *Cistanche* species [12,17,24]. In many cases, phenolics constitute the dominant fraction of characterized metabolites and are frequently associated with antioxidant, antimicrobial, and anti-inflammatory activities observed in vitro. While such activities are well documented for phenolics in general, their prevalence in parasitic plants suggests that they may represent a conserved and functionally important component of their metabolic repertoire. In addition to intrinsic biosynthesis, it has been proposed that some phenolic compounds in parasitic plants may be partially derived from, or structurally influenced by, host metabolites, further complicating the interpretation of their origin and function.

In contrast, alkaloids appear to be less uniformly distributed and more taxon-specific in parasitic plants. Although alkaloids are widely recognized for their potent pharmacological activities—including neuroactive, cytotoxic, and antimicrobial effects—in non-parasitic species [93,94,95], their occurrence in parasitic taxa is comparatively limited. When detected, alkaloids are often restricted to particular genera or species and may occur at lower abundance than phenolic compounds. This uneven distribution suggests that alkaloid biosynthesis may be reduced or selectively retained in certain parasitic lineages, potentially reflecting evolutionary trade-offs associated with parasitic lifestyle. Nevertheless, in species where they are present, alkaloids may contribute disproportionately to biological activity due to their high potency, although this remains insufficiently explored in most parasitic systems.

Terpenoids and related isoprenoid compounds have also been reported in parasitic plants, but their characterization remains relatively limited compared to phenolics. These compounds include mono-, sesqui-, and triterpenes, as well as structurally related metabolites such as sterols and iridoids. In particular, members of the Orobanchaceae family, especially holoparasitic genera such as *Cistanche* and *Orobanche*, are known to contain phenylethanoid glycosides, iridoids, and lignans, which have been associated with antioxidant, neuroprotective, and anti-inflammatory activities [46]. These findings suggest that certain parasitic lineages may possess distinct and potentially valuable metabolite profiles, although systematic comparative studies across taxa remain scarce.

It is also important to consider that the phytochemical composition of parasitic plants may be influenced by host–parasite interactions, including the uptake or modification of host-derived metabolites. This adds another layer of complexity to interpreting pharmacological properties and distinguishing intrinsic biosynthetic capacity from acquired compounds.

A comprehensive overview of biosynthetic pathways and a detailed classification of these metabolite groups are beyond the scope of the present review. Instead, this study focuses on the major classes of secondary metabolites reported in medicinally relevant parasitic plants for which phytochemical and pharmacological data are available (Table 1). Overall, current evidence indicates that polyphenolic compounds dominate the phytochemical profiles of most parasitic species studied. In contrast, other metabolite classes, including alkaloids and bioactive peptides, appear more restricted and species-specific.

## 5. Parasitic Plants as a Source of Unique Bioactive Compounds

Apart from the diversity of common plant metabolites that are also present in parasitic plants, it is important to evaluate whether there is sufficient evidence for bioactive compounds that are unique or highly characteristic of parasitic taxa. Several secondary metabolites have been reported as relatively specific to certain parasitic plant genera. For example, cynomoriitannin has been identified as a characteristic condensed tannin, or proanthocyanidin, in *Cynomorium* [101], composed mainly of catechin and epicatechin residues. Similar compounds, known as procyanidins, are well-characterized polyphenols found in various plant sources and are commonly used as food [102]. Flavonoid glycoside—cuscutin, alkaloid—cuscutamine, together with resin glycosides known as cuscutosides, are associated with species of *Cuscuta* [103]. However, they are relatively poorly studied, have unclear chemical structures, and are most likely similar to other common phytochemicals.

Members of the Orobanchaceae family are particularly rich in phenylethanoid and iridoid glycosides, including acteoside. However, this compound is not exclusive to broomrapes and has also been reported in other parasitic genera such as *Lathraea* [104], as well as many other dicotyledonous plants [105]. Phenylethanoid glycosides, including cistanoside and tubuloside, represent some of the best-characterized bioactive compounds in parasitic plants, particularly within the genus *Cistanche* [106]. These compounds have been extensively studied and are associated with antioxidant, neuroprotective, and anti-inflammatory activities in experimental models.

In addition to secondary metabolites, parasitic plants produce distinctive proteins and peptides with significant biological activity. Cuscutain, a proteolytic enzyme identified in *Cuscuta*, represents one example of such specialized proteins [107]. In mistletoe (*Viscum album*), two major groups of biologically active compounds are considered highly characteristic. Viscotoxins are small cysteine-rich proteins found exclusively in European mistletoe and function as defensive molecules that protect the plant against herbivores and microbial pathogens. These proteins have demonstrated notable pharmacological potential, including anticancer, antimicrobial, and immunostimulatory effects, and are incorporated into mistletoe-based complementary cancer therapies such as Iscador^®^ [108]. Their biological activity includes cytotoxic effects on tumor cells through the induction of apoptosis. Another group of mistletoe-specific bioactive compounds is mistletoe lectins, which are ribosome-inactivating proteins particularly abundant in *Viscum album*. These lectins are believed to play a role in host interaction and possibly in the modulation of host defense responses. Pharmacologically, mistletoe lectins exhibit strong anticancer properties by stimulating immune responses and promoting apoptosis in cancer cells. They have also been investigated for potential therapeutic applications in autoimmune and inflammatory diseases [108].

Overall, current evidence indicates that while parasitic plants contain a wide array of bioactive compounds, truly unique or taxon-specific metabolites remain relatively rare and insufficiently characterized. Many compounds, which are considered characteristic, are structurally and biosynthetically related to widely distributed plant secondary metabolites. This suggests that parasitic plants may rely more on modification, accumulation, or differential regulation of common phytochemical pathways rather than on the evolution of entirely novel chemical entities.

A major limitation in the field is the uneven depth of phytochemical investigation across parasitic taxa. Many reported compounds, especially in genera such as *Cuscuta*, are still poorly defined structurally, lack a comprehensive biosynthetic context, and have not been rigorously compared with homologous compounds in non-parasitic plants. Consequently, claims of specificity should be treated with caution. There is also a lack of in-depth research into the actual transfer of specialized metabolites from the host to the parasitic plant, despite abundant indirect evidence for this process. Future research should prioritize high-resolution metabolomics, comparative genomics, and functional assays to distinguish truly lineage-specific metabolites from broadly conserved compounds. Integrating these approaches will be essential for clarifying whether parasitism drives chemical innovation or primarily reshapes existing metabolic frameworks.

## 6. Safety and Toxicity

As much as any other plant extract, used in pharmaceuticals, cosmetics, and traditional medicine, the safety and toxicity profiles of parasitic plants’ extracts must be carefully evaluated before use. Although often perceived as “natural” and therefore harmless, many plant-derived compounds can exert potent biological effects, including cytotoxic, mutagenic, or allergenic activities. The chemical composition of plant extracts can vary significantly depending on factors such as species, growing conditions, harvesting time, and extraction method [109], a pattern further complicated in parasitic plants by host influence.

The safety profile of *Viscum album* is probably the most considered among parasitic plants due to its application as a complementary anti-cancer therapy. The extracts, containing lectins, viscotoxins, and alkaloids, contribute to its pharmacological effects but also pose a risk of toxicity. Especially lectins and viscotoxins are potent cytotoxic agents, causing substantial safety concerns [110]. Whereas commercial products are standardized and have undergone dose testing, ingestion of raw plant material, particularly the berries, can cause adverse effects, including nausea, vomiting, hypotension, and bradycardia, with severe cases leading to neurological symptoms [111]. To date, several systematic reviews have been published, two of which are specifically dedicated to safety and toxicity: one from 2009 [112] and one from 2011 [110]. In the first, 18 clinical trials of standardized medicinal preparations involving over 6800 patients were analyzed, and no significant adverse effects were found [112]. The second one analyzed 48 animal studies and 69 clinical studies and reported a variety of adverse effects; however, at higher doses, the application of mistletoe extracts was generally considered safe [110]. More recent animal [113] and clinical [114] studies confirmed the overall safety of mistletoe extracts, although it should be stressed that this is mainly valid for commercial standardized preparations.

Unlike *Viscum album* extracts, other parasitic plants were not subjected to extensive safety evaluation in clinical studies but were instead evaluated in animal models or in vitro cultures. Both *Cistanche tubulosa* and *C. deserticola* water or methanolic extracts were tested on rats [115,116,117] and suggested relative safety, although only one of the studies involved a standardized food product—Memoregain^®^ [116].

The toxicological profile of *Cuscuta epithymum* L. was also recently reviewed [16], along with other *Cuscuta* species, and based on several studies of methanolic and ethanolic extracts, the species was concluded to be generally safe. Other recent research on C. chinensis in murine models [118,119] confirmed this, with “safety” defined as an LD50 ranging from 200 to 5000 mg kg^−1^ body weight. However, a single 2013 study reported acute toxicity of C. campestris in horses fed as a contaminant in alfalfa [120], with an abnormal hematological profile and clinical signs. This is not widely reported in the literature, and in this particular case, it is difficult to evaluate, but it should be noted that complete safety cannot be claimed for any product.

Overall, challenges in the safety assessment of parasitic plant extracts arise from a lack of standardization, with a few exceptions, as few commercial preparations are available and parasitic plants are difficult to cultivate. Therefore, most of the extracts are prepared from wild sources with many unknowns (for example, the host identity). In some cases, especially within the subgenus *Grammica* of *Cuscuta*, the visual similarity between species is so close that even the parasitic plant identity could be mistaken [121], leading to dramatic differences in the bioactive compound profile.

## 7. Cultivation of Medicinal Parasitic Plants

Cultivation of parasitic plants sounds contrived at first glance. They are usually associated with their detrimental effect on agriculturally important crops. However, only a few of the 4700 species are significant pests [122]. Besides them, and despite the challenges related to the requirement for a suitable host, the cultivation of parasitic plants is of particular conservation interest. Many parasitic plant species are actually considered rare, or endangered, and their successful propagation is considered of key importance for their conservation. One such example is the genus Rafflesia, most of which are endangered [56] and subject to cultivation and propagation efforts [123,124]. Such efforts might also be needed for medicinal parasitic plants, as wild collection and overexploitation are well-known factors contributing to the population decline of medicinal plants [125,126].

Currently, there are at least two examples of successful cultivation of medicinal parasitic plants. The cultivation of *Viscum album* is due to its importance in cancer therapy. It has been practiced for centuries, primarily for medicinal, cultural, and ecological purposes. Historical records from ancient Greek and Roman sources describe mistletoe as a sacred and medicinal plant. By the Middle Ages, it was intentionally maintained on orchard trees in parts of Europe [127]. Modern cultivation techniques began to develop in the late nineteenth and early twentieth centuries, particularly after mistletoe extracts were investigated for medical use in Central Europe. Cultivation typically involves manually placing ripe berries or seeds onto suitable host tree branches such as apple, oak, poplar, or pine, where the seeds germinate and form haustorial connections with host tissues. Large-scale cultivation efforts expanded during the twentieth century to support pharmaceutical production, especially in Germany and Switzerland, where host-specific mistletoe cultivation became important for producing standardized medicinal extracts. Today, *Viscum album* cultivation continues to support research, traditional medicine, and biodiversity conservation, although successful establishment requires careful host selection and long growth periods [128,129,130].

Cultivation of *Cistanche tubulosa* and *C. deserticola* Ma., holoparasitic desert plants widely used in traditional Chinese medicine, has developed significantly over the past several centuries, particularly in arid regions of China and Central Asia [131,132]. Historical records from traditional Chinese herbal medicine texts dating back over 1500 years describe the plant as a valuable tonic, prompting extensive wild harvesting that led to severe population declines by the late twentieth century [49]. Modern cultivation began to expand in the 1980s and 1990s in response to conservation concerns and rising demand for medicinal use. Because *Cistanche deserticola* is an obligate root parasite, cultivation requires planting host species, most commonly *Haloxylon ammodendron* (C.A.Mey.) Bunge ex Fenzl or *Haloxylon persicum* Bunge, in desert or semi-desert environments. After host establishment, parasite seeds are introduced into the soil near host roots, where germination and haustorium formation occur under suitable environmental conditions [132]. Large-scale cultivation programs are now established in regions such as Inner Mongolia and Xinjiang, supporting both the ecological restoration of desert ecosystems and the sustainable production of medicinal raw materials, while reducing pressure on natural populations [133].

An important aspect to consider in the cultivation of parasitic plants is the standardization of medicinal raw materials. Unlike non-parasitic species, the chemical composition of parasitic plants is strongly influenced by the host, which can significantly affect both the qualitative and quantitative profile of bioactive compounds. For example, in *Viscum album*, host tree species are known to influence lectin and viscotoxin content, necessitating careful host selection to ensure consistent pharmacological properties [134]. This host dependency poses challenges for reproducibility and complicates efforts to achieve uniform chemical profiles across cultivated batches. In addition, large-scale cultivation of obligate parasites, such as *Cistanche deserticola*, remains technically demanding because it requires the simultaneous management of both the host and the parasite under controlled environmental conditions. These factors highlight the need for integrated standardization strategies that combine controlled cultivation systems, chemical profiling, and rigorous quality control to ensure the safety, efficacy, and reproducibility of parasitic plant-derived products.

## 8. Conclusions

Parasitic plants represent a substantial and intriguing source of bioactive compounds, some of which may be unique or exhibit distinct pharmacological properties. However, their evaluation as medicinal resources requires careful consideration of several fundamental aspects. Unlike autotrophic plants, the bioactive profiles of parasitic species arise from a complex interplay between intrinsic metabolic capacity, host-derived compounds, and dynamic host–parasite interactions mediated through haustorial connections. This multi-layered origin of metabolites complicates the attribution of biological activity and challenges the distinction between compounds synthesized de novo and acquired. Although parasitic plants cannot be regarded as universally superior sources of medicinal compounds, certain lineages produce characteristic metabolites and bioactive proteins with demonstrated therapeutic relevance, particularly for anticancer, antioxidant, and immunomodulatory activities. Nevertheless, many of these compounds belong to widely distributed chemical classes, raising questions about the extent to which parasitism contributes to true chemical innovation versus modification and accumulation of existing phytochemicals.

A major limitation in the field is the uneven distribution of research efforts across parasitic taxa, with a strong bias toward a few well-studied genera, while many lineages remain poorly explored. In addition, the pronounced influence of host identity on metabolite composition introduces significant variability, complicating reproducibility, standardization, and pharmacological validation. These factors not only limit comparability between studies but also pose challenges for the development of reliable medicinal products derived from parasitic plants. Future research should therefore adopt integrative, multidisciplinary approaches that combine advanced phytochemical analysis, high-resolution metabolomics, genomics, and functional studies of host–parasite interactions. Particular attention should be given to elucidating the mechanisms of metabolite transfer and transformation, as well as to distinguishing host-derived compounds from those synthesized or modified by the parasite. In parallel, the development of sustainable cultivation systems and conservation strategies will be essential to ensure both the protection of natural populations and the consistent production of high-quality raw materials.

## Figures and Tables

**Table 1:** Major classes of bioactive compounds identified in representative parasitic plant species.

Parasitic Plant Species	Lineage	Compound Class	Compound Subclass	Major Compounds	Pharmacological Activities	Reference
*Viscum album*	Santalales, stem hemiparasites	Polyphenols	Flavonoids	Apigenin, Kaempferol, Quercetin, Naringenin	Antioxidant, Antimicrobial Immunomodulatory, Anti-cancer, Anti-inflammatory	[24,96]
			Phenolic acids	Caffeic acid, Rosmarinic acid, Ferulic acid		
		Peptides	Lectins	ML I-III		
			Viscotoxins	Viscotoxins A1-A3, B, C		
*Cuscuta chinensis*	Solanales, stem holoparasite	Polyphenols	Flavonoids	Kaempferol, Quercetin, Hyperoside	Antioxidant, Antimicrobial Immunomodulatory	[97]
			Phenolic acids	Caffeic acid, Coumaric acid		
		Alkaloids	Quinazoline alkaloids	Cuscutamine, Agroclavine		
		Resin glycosides		Cuscutoside		
		Steroids		Campesterol, Stigmasterol		
*Cistanche tubulosa* (Schrenk) Hook.f.	Lamiales, root holoparasite	Polyphenols	Phenylpropanoid glycosides	Acteoside, Tubuloside	Antioxidant, Anti-inflammatory, Hepatoprotection	[53,98,99]
			Lignans	Pinoresinol		
		Terpenoids	Iridoid glycosides	Cistanin, Cistachlorin, Bartsioside		
*Taxillus chinensis* (DC.) Danser	Santalales, stem hemiparasites	Polyphenols	Flavonoids	Quercetin, Taxifolin, Catechin, Hyperoside	Anti-inflammatory, Antioxidant, Anticancer, Antimicrobial	[100]
			Phenolic acids	Quinic acid, Gallic acid, Coniferic acid		
			Tannins	Glucogallin, Procyanidins		

## Data Availability

No new data were created or analyzed in this study.
